# Monthly Memoranda

**Published:** 1853-10

**Authors:** 


### EDITORIAL DEPARTMENT.

MONTHLY MEMORANDA. — *Conception without Menstruation.*—Dr. Gregory, of Warren, Mo., reports to Dr. Meigs, of Philadelphia, an interesting case, which bears upon this curious question. Dr. G. mentions a patient of his, the mother of six living children, (all boys,) who had never experienced the usual catamenia. Dr. G. attended her at her seventh labor. She stated that she had never been unwell, never experienced the usual attendant symptoms, never had a vicarious discharge, except twice, when she threw up dark grumous blood. Her general health had been good, and she usually attended to her own domestic affairs. Dr. Meigs comments upon this case, "that as fecundation without a previous act of ovulation, is not to be deemed possible, this lady was the subject of the germ-producing act as fully as any other woman can be; and that, as the menstrual discharge is nothing more than a simple physiological effusion of blood, determined by the process of ovulation, it is for many women indifferent whether the sanguineous discharge does not coincide with the oviposit. Probably, menstruation consists, more essentially in the periodical oviposit, than in the bleeding from the womb that attends the passages of that important office. In this case, no visible signs of menstrual hyperæmia have ever presented themselves, save only those we observe as to the conceptions and gestations which are the most characteristic of them all." Dr. Meigs urges to "beware not to found upon these interesting facts a decision as to the question, whether a non-menstruating young woman is marriageable, or not. I have met with too mnny cases of total absence of the uterus in married women, not to feel how necessary it is, in all such difficult cases, to have the facts clearly understood, before a professional sanction is given to a union that cannot but produce unhappiness, where abnormal development by default, renders the marriage hopelessly sterile and the rite impossible.

*Galvanism in Accidents from Lightning.*—A case is reported in the Boston Med. and Surg. Journal, by Dr. Simpson, N. H., of a girl, aged 8 years, struck by lightning. She was prostrated and apparently dead. Cold water was thrown on her and a kind of gasping respiration produced. Dr. S. arrived forty minutes after the shock, when the intervals between the gasping respirations were so long, that each appeared to be the last. The body was cold, no pulse, precordial region still, and only the slightest cardiac impulse to be heard. In this state of things, he applied a powerful galvanic current along the dorsal vertebræ, also warmth and friction to all parts of the body. As soon as the galvanic current passed along her back, respiration and the heart's action improved at once. After the application had lasted ten minutes, and respiration was well established, he discontinued the galvanism, when the gasping state returned, with rattling in the throat and complete relaxation. Again the galvanism was applied, and continued, with slight intermissions, for an hour or more, when she swallowed, though hardly voluntarily. A stimulant was given, which provoked vomiting. After this, appropriate treatment was employed to remove congestions which had ensued, and establish proper nervous action in the lungs, kidneys and bowels. After reaction was fairly established, ordinary treatment availed. This case would seem to indicate that lightning produces death by overpowering the nervous system, and rendering it insensible, and galvanism would seem to be useful treatment, within a reasonable period, say an hour.

*Tartaric Acid aiding the solution of Quinine.*—A few drops of dilute sulphuric acid will, as is well known, aid to dissolve disulphate of quinine, but the taste of the mixture is very bitter and disagreeable. Messrs. Bouchardat, Righni and Ruspini, advise tartaric acid to be used for the purpose of hastening the solution of the quinine, instead of sulphuric acid. It has been found that one grain of tartaric acid will saturate three of the sulphate of quinine, the compound being a sulpho-tartrate of quinine, and not not at all unpleasant to the taste.

*Vesicatories.*—When it becomes necessary to use blisters to inflamed joints, as in some forms of rheumatism, the previous application of a mustard cataplasm will be advisable, as, after full vesication, when there is a redundance of fibrine, the vesicle will be filled with it, and the vessels thereby relieved to a much greater extent than without the previous use of the mustard.

*The Queen of England*, it has been stated and currently *believed, actually* took Chloroform in her late accouchment. *Mirabile dictu!!* This is an astonishing fact and should be heralded from Dan to Beersheba. We are also assured that a *mechanic's lady* in the U. S., took it in the same way, under like circumstances, and with like results save that the latter gave birth to a *sovereign*, while in the former case the offspring was only a *prince* or a *princess*.

*Holme's Liniment for Lumbago.*—Powdered chamomile, 8 parts; common salt, 2 parts; camphor, (previously dissolved in turpentine), 1 part; oat meal, 100 parts; black soap, 30 parts.—*Med. and Surg. Journal, Virginia.*

*Intestinal Obstructions.*—Mr. B. Phillips advises in intestinal obstructions, that the drastic purgatives, such as croton oil, should not be given at an early period; but one or two full doses of calomel and opium (8 to 10 grs. calomel and 2 grs. opium) should first be given, and large emolgient enemata be thrown up, every six or eight hours. If these means fail, Mr. Phillips pushes mercury to salivation; mercurial inunction, as well as by the mouth, being employed.—*British For. Med. Chir. Review.*

In *intestinal obstructions* arising from impacted fœces, in the lower bowels, why not dissolve the ox gall to the amount of 2 scruples in warm water to be used in form of enemata. From its solvent powers it is indicated, and has been tried with success. For the same reason why not administer it in dysentery, where there are concretions in the color. During this season, we administered it in one case of the kind, conjoined with pulv. dover. Three grains of the inspissated ox gall with as many of the Dover's powder were given every four hours during the night, and the next morning, instead of the small muco-sanguineous discharges which had been preceded by termina and attended with tenesmus, she had several large dejections without any suffering whatever. As the case was obstinate up to that time, and, as these concretions were uniformly present, we inferred favorably for this agent. In these cases as we have suspended biliary secretion, does not the Fel. Bov. supply this want to some extent.—*Editors.*

*Epistaxis.*—Powdered gum arabic and alum in equal parts blown up the bleeding nostril has been found in very many cases to arrest the hemorrhage. So successfully has it proved in our hands that we would recommend it confidently to the attention of the profession.—Because of its success in the above mentioned difficulty, we have been induced to make trial of it in uterine hemorrhage. Small squares of muslin, half worn, three inches square were rubbed into the dry powder until the tissue was well filled with it, when they were introduced one after another until the vagina was well filled. The result would justify us in a further resort to it in those cases in which the tampon is indicated.

*The British Parliament* has recently enacted a law, making vaccination compulsory; requiring that every child born in England and

Wales, after the first of August, 1853, must be vaccinated within three months after birth. A neglect of this law, involves a severe fine. This is as it should be. Society has, and should exercise the right to defend itself by all such sanatory regulations, against the violence and even the existence, if possible, of epidemics. No man has a right to maintain such a condition of his property, or even of his person, as may involve hazard to the lives of others. We earnestly hope that the movement which has thus been commenced in England, may be followed in our own country. A law should be enacted, in this country, applying not only to our own native born citizens, but also to all emigrants, for it is in this portion of our communities that almost every form of pestilence finds its richest harvest. The only approach to such a law, of which we are conscious, is a municipal regulation in many Eastern cities, that no pupil can enter a public school, without a certificate of vaccination, from a regular physician. But such local regulations, though in the proper direction, do not go far enough. The community have a right to demand of the State Legislatures, that they be protected, by well executed laws, against the incursion and ravages of such fearful epidemics.

*Colica Pictorum.*—It is stated by Dr. Swett, of N. Y., that the treatment of this disease by strychnia, which he proposed more than a year since, has become the fixed practice of the N. Y. Hospital. He attributes the disease to paralysis of the intestines, and thinks that strychnia relieves it by its peculiar action upon the nerves.——Dr. Watson has been successful in treating lead colic with tobacco. His mode of employing it is to apply a cataplasm of tobacco over the abdomen; relief is usually obtained in less than 48 hours. Our own judgment would be that in many cases, it would be important to watch the effects of so powerful an agent as tobacco.

